# Reliability and Usefulness of Different Biomarkers of Oxidative Stress in Chronic Obstructive Pulmonary Disease

**DOI:** 10.1155/2020/4982324

**Published:** 2020-05-14

**Authors:** Elisabetta Zinellu, Angelo Zinellu, Alessandro G. Fois, Sara S. Fois, Barbara Piras, Ciriaco Carru, Pietro Pirina

**Affiliations:** ^1^Respiratory Unit-Azienda Ospedaliero Universitaria, Sassari, Italy; ^2^Department of Biomedical Sciences, University of Sassari, Sassari, Italy; ^3^Respiratory Unit, Department of Medical, Surgical and Experimental Sciences-University of Sassari, Sassari, Italy

## Abstract

**Introduction:**

Chronic obstructive pulmonary disease (COPD) is a progressive disease characterized by airflow limitation that is not fully reversible after inhaled bronchodilator use associated with an abnormal inflammatory condition. The biggest risk factor for COPD is cigarette smoking. The exposure to noxious chemicals contained within tobacco smoke is known to cause airway epithelial injury through oxidative stress, which in turn has the ability to elicit an inflammatory response. In fact, the disruption of the delicate balance between oxidant and antioxidant defenses leads to an oxidative burden that has long been held responsible to play a pivotal role in the pathogenesis of COPD. There are currently several biomarkers of oxidative stress in COPD that have been evaluated in a variety of biological samples. The aim of this review is to identify the best studied molecules by summarizing the key literature findings, thus shedding some light on the subject.

**Methods:**

We searched for relevant case-control studies examining oxidative stress biomarkers in stable COPD, taking into account the analytical method of detection as an influence factor.

**Results:**

Many oxidative stress biomarkers have been evaluated in several biological matrices, mostly in the blood. Some of them consistently differ between the cases and controls even when allowing different analytical methods of detection.

**Conclusions:**

The present review provides an overview of the oxidative stress biomarkers that have been evaluated in patients with COPD, bringing focus on those molecules whose reliability has been confirmed by the use of different analytical methods.

## 1. Introduction

Chronic obstructive pulmonary disease (COPD) is a major cause of morbidity and mortality worldwide, so much so that it has now become the third leading cause of death by disease [1]. COPD is characterized by a persistent and progressive airflow limitation associated with a chronic inflammatory condition [2]. Cigarette smoking is notably the main cause of COPD, but other factors are being identified at an increasing rate such as occupational exposure, biomass smoke inhalation, and *α*_1_ antitrypsin deficiency [3]. Acute exacerbations and comorbidities contribute to disease severity in individual patients [4]. Tobacco smoke contains 10^17^ oxidant molecules per puff [5]. Exposure to these oxidants causes direct injury to airway epithelial cells leading to airway inflammation. Inhaled particles and mediators of inflammation activate phagocytic cells such as neutrophils and macrophages, which in turn produce large amounts of reactive oxygen species (ROS) that need to be counterbalanced by antioxidant factors [6]. In addition, ROS may react with nitric oxide (NO) produced by the inducible form of nitric oxide synthase during inflammation, to form various oxidant species such as peroxynitrite [7]. When oxidants are produced in excess of the antioxidant defense mechanisms, oxidative stress occurs resulting in harmful effects, including damage to lipids, proteins, and nucleic acids [8]. It is now well established that oxidative stress plays a key role in the pathophysiology of COPD [9–11]. Several biomarkers of oxidative stress have been studied, including ROS themselves. Direct measurement of ROS levels is difficult due to their short lifespan and rapid reactivity; indirect determination by examining oxidation target products is therefore a good alternative approach [12]. As regards COPD, various potential biomarkers of oxidative stress have been evaluated in different biological samples, especially in peripheral blood and exhaled breath condensate (EBC), but also in induced sputum, urine, and to a lesser extent, broncoalveolar lavage fluid (BALF) as well as bronchial or muscle biopsies. In this review, we summarize to the best of our knowledge the main literature findings regarding oxidative stress biomarkers in the abovementioned biological samples in patients with COPD compared to the healthy controls. We have considered and listed our findings based on the method used to detect the biomarkers, because we believe this is likely to be a major influence factor on the results.

## 2. Search Strategy and Eligibility Criteria

We conducted a literature search involving electronic databases such as PubMed, Web of Science, and Scopus from inception to June 2019. The combinations of keywords used were “chronic obstructive pulmonary disease” or “COPD,” “oxidative stress,” and “biomarkers.” All relevant studies were included based on predetermined eligibility criteria. Specifically, we included published case-control studies if (i) they were conducted on human subjects, (ii) there was an assessment of oxidative stress biomarkers, (iii) they were conducted in patients in stable phase of COPD, and (iv) they were written in English.

## 3. Oxidant Biomarkers

### 3.1. Lipid Peroxidation Products

Lipids can be oxidized by various mechanisms that yield different products [13]. These products have received much attention as biomarkers of oxidative stress [14]. The most commonly studied molecules in COPD are malondialdehyde (MDA) and thiobarbituric acid-reactive substances (TBARS) as evidenced in Table 1. The constitution of TBARS requires the reaction of MDA with thiobarbituric acid (TBA), resulting in the formation of a byproduct that can be detected with a spectrophotometer or a chromatograph. The levels of MDA molecules are increased in various biological samples in patients with COPD compared to the healthy controls, and increase further with progression of disease (Table 1) [15–45]. A few studies found no differences using this method of detection [46–54]. Some other studies have investigated MDA using other methods of detection, finding an increase of this biomarker in COPD (Table 1) [55–61].

Other biomarkers of lipid peroxidation such as 8-isoprostane, lipid peroxides, conjugated dienes, oxidized low-density lipoproteins, and ethane have been evaluated in COPD, but to a lesser extent than MDA. These molecules were also increased in COPD compared to the controls (Table 1) [17, 55, 62–70].

### 3.2. Protein Oxidation Products

Byproducts of protein carbonylation, a common variety of protein oxidation, have also been assessed in COPD [71]. The most relevant results on these biomarkers are summarized in Table 1 [19, 25, 26, 30, 41, 50, 55, 56, 72–79]. A widely used method for the determination of carbonyl content is based on the reaction of carbonyl groups with 2,4-dinitrophenylhydrazine (DNPH), which leads to the formation of a stable product that can be detected spectrophotometrically or immunochemically [80]. The majority of studies have described a significant increase in protein carbonyl groups in patients with COPD in various biological samples compared to the healthy controls and in correlation with disease progression. Another object of research is the advanced oxidation protein products (AOPPs). AOPPs are a family of oxidized compounds formed by the reaction of plasma proteins, mostly albumin, with chlorinated oxidants [81]. They have commonly been evaluated in the peripheral blood of COPD patients by means of a microplate spectrophotometer, finding an increase or no difference in the cases versus controls (Table 1) [22, 30, 47].

### 3.3. Reactive Oxygen Species (ROS)

Some authors have explored the level of oxidative stress in COPD patient by looking at the production of ROSs such as superoxide anion (O_2_^−^) and hydrogen peroxide (H_2_O_2_). Detection of O_2_^−^ was based on chemiluminescence assays using a luminometer [82]. Alternatively, enzymatic assays were used to measure the reduction of a substrate by O_2_^−^. Superoxide anion blood levels were found increased in COPD patients compared to the healthy controls [22, 26, 29, 47, 48, 74] (Table 2).

Production of H_2_O_2_ was studied with horseradish peroxidase-containing enzyme assays or by measuring reactive oxygen metabolites (ROMs) with diacron-reactive oxygen metabolites (d-ROM) in EBC. All studies have shown that H_2_O_2_ is increased in the EBC of patients with COPD compared to the controls as well as in relation to disease progression [43, 64, 83–85] (Table 2).

### 3.4. Total Oxidant Status

A few authors have studied the total oxidative status (TOS) in patients with COPD as a marker of oxidative stress. This can be evaluated by surveying the oxidation of a ferrous ion by the oxidants present in the chosen biological sample or by means of the d-ROM test. Total oxidative status was elevated in the blood of COPD patients in all of the examined studies. [22, 86–91] (Table 2).

### 3.5. Oxidatively Damaged DNA

Products derived from DNA oxidative damage have also been examined in connection with COPD. Comet assay, a single-cell gel electrophoresis, has been used in various studies to detect DNA strand breaks. Another commonly used marker for assessing oxidative damage to nucleic acids is 8-oxo-7,8-dihydro-2′-deoxyguanosine (8-oxodG). By means of these methods, a significant increase or no differences in the levels of DNA damage has been detected in the blood and urine of COPD patients compared to the controls; however, a significant increase has been found in sputum. [25, 30, 37, 43, 75, 92–97] (Table 2).

### 3.6. Peroxynitrites and Nitrotyrosines

Peroxynitrite is the product of the reaction of nitric oxide and superoxide radicals; it is a powerful oxidant that promotes tyrosine nitration with formation of nitrotyrosines. Peroxynitrite levels can be determined using oxidation of a fluorescein or by spectrophotometric detection of nitrophenol, a molecule resulting from the nitrating properties of peroxynitrite itself. Protein tyrosine nitration has also been evaluated by means of ELISA or immunocytostaining in sputum cells. Both peroxynitrites and nitrotyrosines have been studied in patients with COPD compared to the controls in various biological samples and by means of a multitude of assays, finding both an elevation and no difference [30, 61, 75–78, 98–102] (Table 2).

## 4. Antioxidant Biomarkers

### 4.1. Protein and Nonprotein Thiols

Thiols are organic compounds containing a sulfhydryl group (–SH). In humans, blood proteins harbor the largest amount of thiol groups, while a smaller proportion is represented by nonprotein thiols such as glutathione (GSH). Thiols can undergo oxidation processes in the presence of oxidants and constitute an important component of the antioxidant defense system. The plasma thiol level is most commonly determined using Ellman's reagent, 5,5′-dithiobis-2-nitrobenzoic acid. This compound is reduced by free thiols in an exchange reaction that releases a product that can be measured with spectrophotometry [103]. In patients with COPD, protein thiols have predominantly been examined in the blood, where a significant reduction compared to the controls has often been discovered; as regards disease progression, both a decrease and no significant difference have been found (Table 3) [19, 20, 26, 29, 30, 36, 38, 51, 52, 54, 104]. Nonprotein thiols, especially reduced GSH, have been investigated in various biological samples of patients with COPD using different assays. Results have often demonstrated a reduction of this marker compared to the controls and in a few cases, an elevation or no difference at all (Table 3) [16, 18, 19, 24, 26, 27, 30, 31, 33, 35, 40, 42, 46, 49, 51, 52, 72, 96, 105–107].

### 4.2. Total Antioxidant Capacity

Many researchers have investigated total antioxidant capacity in the blood and broncoalveolar lavage fluid of patients with COPD using different assays, particularly the FRAP (ferric-reducing ability of plasma) [108] and the TEAC (Trolox equivalent antioxidant capacity) assays [109]. The largest proportion of these studies has highlighted a decrease of this biomarker in COPD that positively correlates with disease progression (Table 3) [19, 21, 22, 26, 30, 39, 47, 53, 87, 89, 104, 110–113].

### 4.3. Antioxidant Enzymes

Antioxidant enzymes are an important component of the antioxidant defense system. Some of them have been evaluated in patients with COPD to assess oxidative stress. A few studies have focused on these biomarkers analyzing the sputum, BALF, bronchial biopsies, diaphragm or quadricep muscle biopsies, and above all, peripheral blood samples. Most studies concentrated on specific antioxidant enzymes such as superoxide dismutase (SOD), catalase, glutathione peroxidase (GSHPx), and to a lesser extent, glutathione-S-transferase (GST) and paraoxonase 1 (PON1).

SOD catalyzes the dismutation of superoxide anion O_2_^−^ to hydrogen peroxide H_2_O_2_, which is subsequently detoxified to oxygen and water by other enzymes. The majority of research work on SOD has so far utilized the assay method developed by Mc Cord and Fridovich [114]. This is an enzymic function where the superoxide anion generated by xanthine and xanthine oxidase reacts with a tetrazolium salt to form red formazan dye, which is then used as a flag detector. SOD inhibits the formation of the formazan dye, and the activity is measured as percent inhibition. Other assays can measure SOD activity using its ability to inhibit other reactions such as the autooxidation of epinephrine to adrenochrome, the autooxidation of pyrogallol, and the formation of nitrite from the reaction of O_2_^−^ with hydroxylamine hydrochloride [115–117]. In other instances, SOD protein levels have been determined using an ELISA kit. Superoxide dismutase has been by far the most studied among antioxidant enzymes biomarkers in COPD and also the one that presented with the most varied results, having been found diminished, increased, or similar to the controls in different studies (Table 4) [16, 17, 19, 20, 25, 26, 30, 31, 40–42, 57, 72, 74–78, 102, 111–113, 118–120]. Catalase is involved in the detoxification of H_2_O_2_ to molecular oxygen and water. Its activity has been measured in COPD by monitoring of the decomposition rate of H_2_O_2_ at the spectrophotometer, and its levels have been estimated with ELISA kits. Results have shown a reduction as well as no difference in different biological samples of patients with COPD (Table 4) [16, 17, 19, 23, 24, 26, 30, 33, 40–42, 61, 75–77, 102, 111, 112, 118, 120]. GSHPx converts reduced GSH to oxidize glutathione (GSSG) while reducing organic peroxides or H_2_O_2_. Spectrophotometry has been used to determine GSHPx activity by direct evaluation of the content of reduced GSH or by metering the oxidation of nicotinamide adenine dinucleotide phosphate (NADPH), a coenzyme that reduces the GSSG formed in the abovementioned reaction. As opposed to superoxide dismutase, studies on GSHPx activity have produced much less conflicting results and have consistently described a reduction of this biomarker in COPD and also in correlation with disease severity (Table 4) [16, 17, 19, 23, 24, 26, 30, 31, 33, 40, 41, 57, 72, 111, 113, 118, 121]. Glutathione-S-transferase catalyzes the formation of glutathione-S conjugates between GSH and certain electrophilic substrates. It has been studied using 1-chloro-2,4-dinitrobenzene as an artificial substrate or investigating the content of isoenzymes by western blot analysis. PON1, an esterase associated with high-density lipoproteins (HDL), protects against the toxicity of some organophosphates and contributes to the antioxidant protection conferred by HDL on low-density lipoprotein oxidation. Its activity has been evaluated in COPD using a two-substrate (paraoxon/diazoxon) activity method or by the hydrolysis of paraoxon alone. GST and PON1 activities have been studied to a minor extent, and a reduction or no difference was described in COPD compared to the controls (Table 4) [22, 34, 38, 54, 55, 57, 86, 111, 122].

### 4.4. Antioxidant Elements

Various antioxidants elements have been measured in COPD by means of spectrophotometric or chromatographic methods: vitamin A, C, and E and *α*- and *β*-carotenes that contribute to the antioxidant defense system; essential trace elements that play a role in oxidant/antioxidant pathways; and uric acid, a powerful antioxidant protecting lipoproteins from oxidation and acting as a scavenger of oxygen radicals. These biomarkers have especially been studied in the blood of patients with COPD, where a reduction or no difference was found compared to the healthy controls [15, 17, 18, 20, 30, 34, 46, 47, 62, 72, 92, 94, 96, 110, 123].

## 5. Conclusions

This review summarizes the main findings on biomarkers of oxidative stress in patients with stable COPD compared to healthy individuals and in relation to disease progression. The most studied biological sample in this context is the peripheral blood, probably because it is an easily accessible source of information and it allows repeated measurements. Urine test and exhaled breath condensate analysis are other noninvasive monitoring tools, even if EBC analysis is indeed difficult to standardize [124]. Sputum induction, on the other hand, presents some degree of invasiveness, and sampling repetition at regular intervals has been reported to produce conflicting results [125]. As regards BALF and bronchial or muscles biopsies, their invasiveness makes repeated measurements very limited.

Numerous biomarkers of oxidative stress have been investigated in the blood, particularly lipid peroxidation products and protein carbonyls. The majority of these studies have reported an increase of these biomarkers in patients with COPD compared to the healthy controls and sometimes in relation to disease progression. Superoxide anion and total oxidative statuses have always been found to be increased in the blood in the examined case-control studies, even if different assays have been used to investigate them. Other oxidant biomarkers such as oxidatively damaged DNA, peroxinitrites, and nitrotyrosines were increased in some studies, but with no difference between the cases and controls in other research works. As regards antioxidant markers, protein and nonprotein SH groups have provided different results. Nevertheless, most studies have reported a reduction in plasma protein SH groups and reduced GSH. As for total antioxidant capacity, most of the studies examined have consistently reported a significant decrease of this parameter in COPD using various methods of analysis. Conversely, the analysis of blood antioxidant nutrient levels such as vitamins A, C, and E and the analysis of enzymatic antioxidant activities in blood have given conflicting results, being found sometimes decreased, and then again sometimes similar to the controls. Fewer markers have been studied in exhaled breath condensate compared to the blood, particularly oxidant markers such as H_2_O_2_, 8-isoprostane, malondialdehyde, ethane, and peroxynitrites. All of them have been reported to be increased in COPD compared to the controls, except MDA for which not all studies have shown an elevation. In urine, isoprostanes, MDA, and 8-oxodG have always been found increased in COPD, so is the case of the oxidant markers MDA, 8-isoprostane, nitrotyrosines, and 8-oxodG in induced sputum. In BAL, elevated nitrotyrosine levels and reduced GSH levels have been described, while no differences have been observed for antioxidant markers. A few studies have considered bronchial biopsies and muscle biopsies, finding an increase in some oxidant markers such as protein carbonylation, lipid peroxidation, and 3-nitrotyrosine. Conversely, conflicting results have been found in some enzymatic activities.

The underlying reason for the conflicting findings of some studies could be attributed to certain variables that must be taken into account during the sample preparation. These variables that are difficult to control can influence the measurements and therefore the results. We must also consider the human biological variation that inevitably affects any clinical study that is conducted in different populations. Lifestyle variables such as nutrition, smoking, and physical activity can also influence the imbalance between oxidants and antioxidants. Nonetheless, despite these complexities, the different methods used, and the variety of biological samples, the present review highlights that various researchers actually share a few concordant results on this topic. Indeed, it is clear that COPD patients do present oxidative stress, showing higher levels of oxidants, especially lipid and protein oxidation products, and diminished antioxidant defenses, particularly protein SH groups, reduced GSH, and total antioxidant capacity, compared to healthy individuals. Suitable biomarkers to accurately diagnose COPD and to monitor its progression and its response to therapy have not yet been identified. In this context, this review provides a full picture of the oxidative stress biomarkers that have so far been evaluated in patients with COPD, highlighting those whose reliability is confirmed in different biological samples applying various analytical methods. There is no doubt that further research is needed for better validation of these markers in well-characterized populations.

## Figures and Tables

**Table 1:** Most relevant findings about lipid and protein oxidation biomarkers.

Specimen	Method of detection	Healthy vs. COPD	In COPD stages
**MDA**			
Blood	Spectrophotometry following reaction with TBA	↑ [15–35, 38–40]/nd [46–54]	↑ [16, 19, 38, 40–42]/nd [30]
	HPLC following reaction with TBA	↑ [36, 37]	
	Spectrophotometry following reaction with methyl-phenylindole	↑ [55–57]	↑ [55, 56]
	HPLC	↑ [58, 59]	

Urine	HPLC following reaction with TBA	↑ [43]	

EBC	LC-MS following derivatization with DNPH	↑ [60]	
	HPLC following reaction with TBA	↑ [44]/nd [45]	↑ [44]

Sputum	LC-MS following derivatization with DNPH	↑ [60]	
	HPLC following reaction with TBA	↑ [45]	
	Spectrophotometry following reaction with TBA	↑ [31]	

Diaphragm muscle biopsies	HPLC	↓ [61]	

**8-Isoprostane**			
Blood	Specific enzyme immunoassay	↑ [63]	

Urine	GC/MS assay	↑ [69]	
	Enzyme immunoassay	↑ [70]	

EBC	Enzyme immunoassay	↑ [63–66]	↑ [66]/nd [64]
Sputum	Enzyme immunoassay	↑ [68]	

**Lipid peroxides**			
Blood	Spectrophotometry using solution containing cholesterol-iodide	↑ [55]	
	Spectrophotometry following reaction with peroxidase	↑ [62]	

**Coniugated dienes**			
Blood	Spectrophotometry	↑ [17, 55]	

**Oxidized LDL**			
Blood	Specific enzyme immunoassay	↑ [62]	

**Ethane**			
EBC	Gas chromatography	↑ [67]	

**Protein carbonyls**			
Blood	Spectrophotometry following reaction with DNPH	↑ [19, 25, 26, 30, 55, 56, 72]/nd [73]	↑ [55]/nd [19, 26, 30]
	Immunochemistry following reaction with DNPH	↑ [41, 74, 75]/nd [76]	
	Selective radioactive labeling with tritiated borohydride	↑ [50]	↑ [50]

Diaphragm muscle biopsies	Immunochemistry following reaction with DNPH	↑ [77]	

Quadriceps muscle biopsies	Immunochemistry following reaction with DNPH	↑ [76, 78, 79]	

**AOPP**			
Blood	Spectrophotometer	↑ [22, 30]/nd [47]	nd [30]

↑ indicates increased levels; ↓ indicates decreased levels; nd: no difference.

**Table 2:** Most relevant findings about other oxidative biomarkers.

Specimen	Method of detection	Healthy vs. COPD	In COPD stages
**Reactive oxygen species (O2** ^−^ ** in blood and H**_**2**_ **O**_**2**_ ** in EBC)**			
Blood	Chemiluminescence	↑ [47, 48, 74]	
	Enzymatic assays	↑ [22, 26, 29]	

EBC	Enzymatic assays	↑ [43, 64, 83]	↑ [43, 64, 83]
	d-ROMs test exhalation kit	↑ [85]	

**Total oxidant status**			
Blood	Determination of ferrous iron (Fe2+) by colorimetric methods	↑ [22, 86–89]	
	d-ROMs test	↑ [90, 91]	

**Oxidatively damaged DNA**			
Blood	Comet assay	↑ [25, 30, 92]	
	Quantification of 8-oxodG with ELISA	nd [75, 93]	
	Quantification of 8-oxodG with HPLC	nd [37]	

Urine	Quantification of 8-oxodG with ELISA	↑ [43, 94]/nd [96]	
	Quantification of 8-oxodG with HPLC-MS	↑ [95]	↑ [95]
	Quantification of guanine-derived products with LC-MS/MS	↑ [97]	

Sputum	Quantification of 8-oxodG with ELISA	↑ [93]	

**Peroxinitrite**			
Blood	Evaluation of nitrophenol formation	nd [30]	

EBC	Oxidation of a fluorescein	↑ [98]	

**Tyrosine nitration**			
Blood	ELISA	nd [76]	

Sputum	Immunocytostaining with antisera	↑ [99]	
	HPLC	↑ [100]	

BALF	ELISA	↑ [101]	

Bronchial biopsies	ELISA	nd [75]	

Diaphragm muscle biopsies	Immunochemistry	nd [61, 77]	

Quadriceps femoris muscle biopsies	Immunochemistry	↑ [76, 78, 102]	

↑ indicates increased levels; ↓ indicates decreased levels; nd: no difference.

**Table 3:** Most relevant findings about nonenzymatic antioxidant biomarkers.

Specimen	Method of detection	Healthy vs. COPD	In COPD stages
**Protein SH groups**			
Blood	Ellman's assay	↓ [19, 20, 26, 38, 54, 104]/nd [29]	↓ [38]/nd [19, 26]
	Quantification of albumin via chemistry automated analyzer	↓ [36]	
	Subtraction of GSH from total thiols	↓ [51]/nd [30, 52]	

**Reduced GSH**			
Blood	Ellman's assay in plasma	↓ [18, 24, 30, 31, 33, 35, 51, 52]/nd [33]	nd [30]
	Ellman's assay in whole blood	↓ [40, 96]	nd [40]
	Ellman's assay in erythrocytes	↓ [16, 27]	↓ [16]
	Colorimetric determination in erythrocytes	nd [46]	

Sputum	Subtraction of oxidized glutathione from total GSH	nd [105]	
	Colorimetric determination	↓ [31]	

BALF	Reaction with Ellman's reagent in plate reader	↓ [106]	

**Total GSH**			
Blood	Reaction with Ellman's reagent and glutathione reductase in plasma		nd [42]
	Reaction with Ellman's reagent and glutathione reductase in whole blood	↑ [19, 26]	↑ [19]

Sputum	Spectrophotometry following reaction with Ellman's reagent and glutathione reductase	↑ [105, 107]	

**Total thiols**			
Blood	Reaction with Ellman's reagent in plasma	↓ [49]/↑ [72]	↓ [30]

**Total antioxidant capacity**			
Blood	FRAP assay	↓ [19, 26]	↓ [19, 26, 111]
	TEAC assay	↓ [30, 39, 53, 104, 110–112]^/^nd [47, 89]	nd [30]
	Assay based on preventing oxidation of orthodianisidine molecules by hydroxyl radicals	↓ [21, 22, 87]	

BALF	Spectrophotometric monitoring of inhibition of ABTS radical formation by antioxidants	nd [113]	

↑ indicates increased levels; ↓ indicates decreased levels; nd: no difference.

**Table 4:** Most relevant findings about enzymatic antioxidant biomarkers.

Specimen	Method of detection	Healthy vs. COPD	In COPD stages
**SOD activity/levels** ^∗^			
Blood (erythrocytes)	McCord and Fridovich assay	↑ [26, 72]/↓ [40, 111, 112]^/^nd [23]	↓ [40]/nd [41]
	Inhibition of epinephrine autooxidation	↓ [16]/↑ [17]	
	Inhibition of pyrogallol autooxidation	↓ [19]/nd [30]	
	Inhibition of nitrite formation by superoxide radical	↓ [20]	

Blood (plasma)	McCord and Fridovich assay	↑ [57]/↓ [31, 120]/nd [74, 75]	nd [42]
	^∗^ELISA	↓ [118]^/^nd [119]	

Sputum	McCord and Fridovich assay	↓ [31]	
	^∗^ELISA	↑ [119]	nd [119]

BALF	McCord and Fridovich assay	nd [113]	

Bronchial Biopsies	McCord and Fridovich assay	nd [75]	

Diaphragm muscle biopsies	^∗^Immunochemistry	nd [77]	

Quadriceps muscle biopsies	McCord and Fridovich assay	↑ [76, 78]	
	^∗^Immunochemistry	↑ [102]^/^nd [76]	

**Catalase activity/levels** ^∗^			
Blood (erythrocytes)	Measurement of H_2_O_2_ decomposition rate	↓ [16, 19, 40, 111, 112]/nd [17, 23, 26, 30]	↓ [16, 40]/nd [30, 41]

Blood (plasma)	Measurement of H_2_O_2_ decomposition rate	↓ [24, 33, 120]/nd [42]	
	Peroxidatic function of catalase assay	nd [75]	
	^∗^ELISA	nd [118]	

Bronchial biopsies	Peroxidatic function of catalase assay	nd [75]	

Diaphragm muscle biopsies	Measurement of H_2_O_2_ decomposition rate	↑ [61]	↑ [61]
	^∗^Immunochemistry	nd [77]	

Quadriceps muscle biopsies	Peroxidatic function of catalase assay	nd [76]	
	^∗^Immunochemistry	nd [76, 102]	

**GSHPx activity/levels**			
Blood (erythrocytes)	Quantification of oxidation of NADPH	↓ [16, 17, 19, 23, 26, 40, 111]	↓ [40, 41, 121]
Blood (plasma)	Quantification of oxidation of NADPH	↓ [24, 31, 33]/↑ [26, 118]/nd [57]	
	Quantification of reduced glutathione	↑ [30]	

Whole blood	Quantification of oxidation of NADPH	↓ [72]	

Sputum	Quantification of oxidation of NADPH	↓ [31]	

BALF	Spectrophotometry following reaction with Ellman's reagent	nd [113]	

**GST activity/levels** ^∗^			
Plasma	Reaction with 1-chloro-2,4-dinitrobenzene	↓ [111]^/^nd [57]	

Sputum	^∗^Western analysis	↑ [122]	

**PON1 activity/levels**			
Plasma	Paraoxon/diazoxon	nd [22, 86]	
	Paraoxon alone	↓ [34]/nd [38, 54]	↓ [55]

↑ indicates increased levels; ↓ indicates decreased levels; nd: no difference.
